# The use of predictive fall models for older adults receiving aged care, using routinely collected electronic health record data: a systematic review

**DOI:** 10.1186/s12877-022-02901-2

**Published:** 2022-03-16

**Authors:** Karla Seaman, Kristiana Ludlow, Nasir Wabe, Laura Dodds, Joyce Siette, Amy Nguyen, Mikaela Jorgensen, Stephen R. Lord, Jacqueline C. T. Close, Libby O’Toole, Caroline Lin, Annaliese Eymael, Johanna Westbrook

**Affiliations:** 1 Centre for Health Systems and Safety Research, Australian Institute of Health Innovation, Macquarie University, Level 6, 75 Talavera Road, Sydney, NSW 2109 Australia; 2 The MARCS Institute for Brain, Behaviour and Development, Western Sydney University, Sydney, Australia; 3 St Vincent’s Clinical School, Medicine, University of New South Wales, Sydney, Australia; 4 Neuroscience Research Australia, Sydney, Australia; 5 School of Public Health and Community Medicine, University of New South Wales, Sydney, Australia; 6 Aged Care Quality and Safety Commission, Sydney, Australia

**Keywords:** Older adults, Falls, Aged care, Predictive modelling, Fall risk, Health informatics, Information technology, Health & safety, Quality in health care, Risk management

## Abstract

**Background:**

Falls in older adults remain a pressing health concern. With advancements in data analytics and increasing uptake of electronic health records, developing comprehensive predictive models for fall risk is now possible. We aimed to systematically identify studies involving the development and implementation of predictive falls models which used routinely collected electronic health record data in home-based, community and residential aged care settings.

**Methods:**

A systematic search of entries in Cochrane Library, CINAHL, MEDLINE, Scopus, and Web of Science was conducted in July 2020 using search terms relevant to aged care, prediction, and falls. Selection criteria included English-language studies, published in peer-reviewed journals, had an outcome of falls, and involved fall risk modelling using routinely collected electronic health record data. Screening, data extraction and quality appraisal using the Critical Appraisal Skills Program for Clinical Prediction Rule Studies were conducted. Study content was synthesised and reported narratively.

**Results:**

From 7,329 unique entries, four relevant studies were identified. All predictive models were built using different statistical techniques. Predictors across seven categories were used: demographics, assessments of care, fall history, medication use, health conditions, physical abilities, and environmental factors. Only one of the four studies had been validated externally. Three studies reported on the performance of the models.

**Conclusions:**

Adopting predictive modelling in aged care services for adverse events, such as falls, is in its infancy. The increased availability of electronic health record data and the potential of predictive modelling to document fall risk and inform appropriate interventions is making use of such models achievable. Having a dynamic prediction model that reflects the changing status of an aged care client is key to this moving forward for fall prevention interventions.

**Supplementary Information:**

The online version contains supplementary material available at 10.1186/s12877-022-02901-2.

## Background

Falls are one of the greatest concerns for older adults globally, with one in four people aged 65 years and over experiencing a fall each year [1]. The incidence of falls increases exponentially with age and frailty level and the consequences can be substantial to the individual and healthcare system [2]. Falls may result in serious physical harm or death [3], can have enduring detrimental effects on older adult mental health (i.e. fall-related anxiety and loss of confidence), and have been found to reduce quality of life [4]. For adults aged 65 years and over in Australia, falls are the largest contributor to injury-related hospitalisations (42%), and have an estimated recurrent health service expenditure of AUD$3.9 billion dollars nationally [5]. In addition to these direct costs, the subsequent indirect costs of loss of income or additional carer burden is also substantial. Those who live in residential (long-term) aged care homes (also known as nursing homes or care homes, internationally), or receive services from home-based or community care providers, are particularly vulnerable, with six out of seven people who suffer fall-related injuries residing in these settings [5]. The ability for aged care service providers to readily identify older adults at risk of first or subsequent falls could assist in preventing such adverse events and reduce the associated negative impacts on health and quality of life.

Falls causing harm are often avoidable and fall prevention is a national safety and quality priority for the Australian healthcare system. Evidence-based best practice guidelines and harm minimisation plans have been developed to improve outcomes specific to Australian hospitals, community, and residential aged care settings. These guidelines provide standardised advice on fall prevention strategies, management procedures for common risk factors, injury minimisation, and responding to falls (including post-fall follow-up) [6]. Promising evidence for reducing fall incidence in frail older people includes multifactorial interventions which look for modifiable fall risk factors and tailor interventions based on the risk factors identified (e.g., balance gait problems, poor vision, weakness, use of mobility aids, dizziness, presence of certain comorbidities, and sub-optimal medication use) [6–10].

Multiple assessment tools to predict falls have been designed and applied to older adults accessing home-based and residential aged care [11]. One of the most commonly used tools is the Falls Risk Assessment Tool (FRAT) [6, 11]. Usually these types of fall risk assessments are completed intermittently: generally on admission; when there is a noticeable health deterioration; routinely every 3–6 months; or after a fall has occurred [6]. However, due to the complexity and inter-play of risk factors, and the potential for individual variation, even on a daily basis, an older adults’ falls risk does not remain static. Thus, while existing fall risk tools may capture relevant information, their value is limited if that information is not contemporary. Electronic health records (EHRs) provide an avenue through which comprehensive and real-time information about individuals can be accessed and present an opportunity to address this need for dynamic fall risk assessments. To the best of our knowledge, in the existing literature, there is a lack of a systematic review that investigates predictive models using EHRs in aged care.

Aged care providers are replacing paper record systems with electronic systems. These electronic systems support improved documentation efficiency and quality, increased legibility and access to multiple users, and often reduce the need for duplicate data collection [12]. The integration of multiple types of older adult’s in aged care data into a single comprehensive EHR provides ready access to contemporary information about a resident’s health care and risks. This potential can be actualised further by the development of predictive models and algorithms which draw on data about risk factors within a resident’s record to perform real-time assessments of falls risk. A predictive model in healthcare is defined as the use of available data to predict the occurrence of a health state or outcome that has not yet been observed [13, 14]. Predictive models generally combine multiple predictors by assigning a weight to each predictor to produce a probability or risk score [14]. For example, EHR data has been used in wide and deep machine learning to predict the onset of type 2 diabetes [15]. In other studies, individual risk scores were calculated for earlier prediction of outcomes including mortality in patients with severe COVID-19 [16]; and clinical data across 70 hospitals were used to develop and validate a predictive model which identified patients in hospital at high risk of readmission early during their stay [17]. Compared to traditional modelling such as regression modelling which seeks to explain association, predictive models require unique consideration for development, validation and updating [13].

EHR data have been used to develop predictive models for fall risk identification and decision support in acute and primary care [18–22]. However EHR uptake of information technology in the aged care sector has been slower. We sought to determine the current evidence base for the design and use of models for predicting risk of falls utilising routinely collected electronic health data in home-based, community and residential aged care settings. Specifically, we aimed to identify i) how fall risk models have been developed; ii) their accuracy and use in fall prediction; and iii) how they have been implemented to prevent falls.

## Method

The planning and the reporting of this review followed the PRISMA guidelines [23]. A completed PRISMA checklist can be found in Additional 1 and a study protocol can be found in Additional 2. The study was registered with PROSPERO (ID = CRD42020198996).

### Information sources and search strategy

The search strategy was developed by the research team in consultation with a clinical librarian. The databases searched included Cochrane Library, CINAHL, MEDLINE, Scopus, and Web of Science. A search was undertaken using the search terms (“Assisted living facilit*” OR “Community care” OR “Elder care” OR “Home care” OR “Housing for the elderly” OR “Long-term care” OR “Nursing care facilit*” OR “Nursing home*” OR “Old age home” OR “Older adult*” OR “Residential care” OR “Residential facilit*” OR “Skilled nursing facilit*”) AND (Fall*) AND (“model*” OR “predict*” OR “algorithm” OR “screen*”). A full outline of search strategies, including database-specific MeSH terms and keywords can be found in Additional 3. All collected studies were merged in the reference manager EndNote Version 9 [24]. Duplicates were removed before conducting title, abstract, and full-text screening. Manual searching of reference lists for included was conducted and snowballed articles and relevant titles flagged. Relevant titles and abstracts were then reviewed against the inclusion criteria with relevant studies included in the synthesis. Full-texts of included abstracts were independently assessed, using the same inclusion criteria with the addition of the setting criterion, by two reviewers (KS, KL).

### Eligibility: Inclusion and exclusion criteria

Predefined eligibility criteria were used to determine the inclusion of abstracts and full-text articles. The eligible population included adults aged 65 years and older. The outcome for the predictive models was a fall. Articles were assessed against the following inclusion criteria: 1) English-language, 2) Peer-reviewed journal article, 3) Full-text available, 4) Utilised a predictive risk model with routinely collected EHRs, and 5) Any quantitative study design (e.g., observational, randomised-control trial). Studies were excluded if they developed a static (once-only) measure of fall risk, or if they were derived from sensor monitoring. A predictive model was defined as a statistical procedure for assigning an individual a probability of developing a future adverse outcome in a given time period. Our review specifically focused on the use of real-time, routinely collected data to predict falls. Due to the recent application of information technologies and predictive models in aged care, we limited our date range to published papers from 2000 onwards. A further inclusion criterion was added at the full-text stage screening: the study involved older adults in residential, community, or home-based care settings. Articles were excluded if falls occurred in an acute or rehabilitation setting. This criterion was added at a later stage as it was often difficult to assess the setting from study abstracts.

### Selection and data collection processes

Title and abstract screening were conducted in Rayyan [25], a mobile and web-based application for systematic reviews, to identify studies that met the inclusion criteria. This step was conducted by four reviewers (KS, KL, CL, AE). The two lead reviewers (KS, KL) made the final decisions during the screening process on abstracts to be included. To ensure inter-rater reliability, a 5% blinded review was conducted between KS and KL, resulting in an agreement rate of 98.9% and an interrater reliability Cohen’s kappa of 74.45% (substantial) [26]. Weekly discussions were held between the four reviewers about articles that were ambiguous in relation to the inclusion criteria. During abstract and title screening, relevant articles that did not meet the inclusion criteria (e.g., systematic reviews), were noted for snowballing purposes.

### Methodological quality assessment of included studies

Critical appraisal of included articles was independently performed by two investigators (KS, NW) via the Critical Appraisal Skills Program (CASP) checklist for Clinical Prediction Rule Studies [27]. This tool was specifically developed for evaluating the quality of predictive modelling studies. It consists of 11 questions across three sections: are the results of the study valid (Section A), what are the results (Section B) and will the results help locally (Section C). The response to each question is either ‘yes’, ‘can’t tell’ or ‘no’. For an article to pass the CASP checklist overall, or any of the three sections, it needs more than 50% of the responses to be a ‘yes’. A third investigator (AN) mediated appraisal discrepancies between the two investigators. No study was excluded due to poor quality.

### Data items, extraction and synthesis

One of the investigators (LD) independently extracted the data to a purpose-designed Microsoft Excel 2016 spreadsheet, which was then verified by two investigators (KS, KL). The following data items were extracted: Author, year, country, setting, population, data source (what electronic record the information was extracted from), study design, statistical model used, deviation and validation cohort sizes, outcome of model, fall rate, number of falls, risk score creation, and the area under the curve for the validation cohort. The predictors included in the models were tabled and categorised based on the investigators’ (KS, KL, JS) clinical and practical experience. Due to the heterogeneity of the articles, a narrative synthesis was conducted to describe similarities and differences between the included articles [28].

## Results

### Study selection

**Fig. 1:**
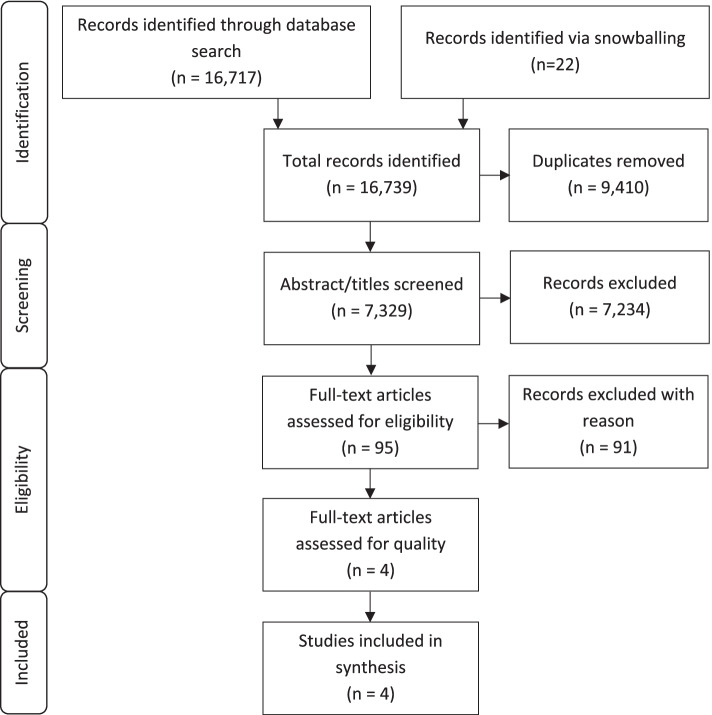
Study selection process (PRISMA), adapted from Page et al. (2021) [23]

The search identified 16,717 entries from the selected databases and a further 22 entries from snowballing techniques. After removal of duplicates, 7,329 articles were screened for title and abstract. In total, 95 full-text articles were retained for further evaluation and four articles met the inclusion criteria (see Fig. 1). The reasons for exclusion were: did not include a predictive risk model (*n* = 71), different setting (*n* = 10), not empirical research (*n* = 4), duplicates (*n* = 3), wrong study type (*n* = 2) and wrong population (*n* = 1).

### Characteristics of included studies

Characteristics of the included studies are provided in Table 1. Three studies came from the United States [29–31] and one from Canada [32]. Two studies were conducted in long-term care facilities [30, 31] and two in home care [29, 32]. Three of the studies were published in the last five years [29, 30, 32] and the other was published in 2005 [31]. Three of the studies were retrospective [30–32] and one was prospective [29]. None of the four studies had implemented the predictive model into practice [29–32].

**Table 1:** Study characteristics

Authors; Year; Country	Setting	Population aged group	Data Source	Study Design	Statistical Model	Derivation Cohort (% of total cohort)	Internal Validation Cohort (% of total cohort) External Validation Cohort	Falls Outcome Prediction	Falls Rate (%)	Risk Score/ Category	Number of models	Discrimination (AUC), (95% CI)	Implemented in practice
Volrathongchai, et al.; 2005; US [31]	Residential Care	65–100 years	MDS	Retrospective	LBP	9,980 (100%)	NR	Fall within 3-months	NR	No	1	NR	No
Marier, et.al.; 2016; US [30]	Residential Care	NR	EMR and MDS	Retrospective	Repeated events survival model	2,527 (49.3%)	2,602 (50.7%)	NR	2.3–32.3% across the deciles in validation cohort depending on the model used	Yes	4 1:MDS Assessments 2: MDS Assessments & EMR only 3: MDS assessments & EMR duplicates 4: MDS assessments & EMR Only & EMR Duplicates	AIC 1: 6733 2: 6749 3: 6614 4:6626	No
Kuspinar, et al.; 2019; Canada [29]	Home care	77 ± 14 years with no previous fall in the last 90 days	RAI-HC	Prospective	Decision tree	88,690 (70%)	Internal: 38,013 (30%) External: 2,738 1,226 9,566	NR	5–35% across risk categories in derivative cohort	Yes	1	NR	No
Lo, et al.; 2019; US [32]	Home care	65 + years	OASIS and EHR	Retrospective	Random Forest Algorithm	29,514 (50%)	29,514 (50%)	NR	5.14% (for emergency care or hospitalisation)	No	3 – Validated against the MAHC-10 1: Combined 2: OASIS 3: MACH model	1: 0.67 2: 0.67 (0.66, 0.68) 3: 0.6 (0.59,0.62)	No

*MDS* Minimum Data Set, *LBP* Likelihood Basis Pursuit, *EMR* Electronic Medical record, *RAI-HC* Resident Assessment Instrument-Home Care, *OASIS* Outcome and Assessment information Set, *AUC* Area under the curve, *AIC* Akaike Information Criteria, *MACH-10* Missouri Alliance for Home Care fall risk assessment, *NR* Not reported

### Quality assessment

Three [29, 30, 32] of the four studies passed the CASP tool criteria based on overall percentage of ‘yes’ responses (score = 54.5%-72.7%). One study [31] did not pass this criteria (score = 27.3%), but was included in the synthesis of data (Table 2). Three out of four studies had a validation cohort [29, 30, 32], however, only one study was externally validated [29]. Two studies had good applicability of their findings to the broader aged care setting [29, 30].

**Table 2:** Critical Appraisal Skills Program checklist

Section ^a^	Study	Volrathongchai et al. (2005) [31]	Marier et al. (2016) [30]	Kuspinar et al. (2019) [29]	Lo et al. (2019) [32]
A	1. Is the Clinical Prediction Rule clearly defined?	Yes	Yes	Yes	Yes
A	2. Is the population from which the rule was derived included an appropriate spectrum of patients?	Yes	Yes	Yes	Yes
A	3. Was the rule validated in a different group of patients?	No	No	Yes	No
A	4. Were the predictor variables and the outcome evaluated in a blinded fashion?	No	No	No	No
A	5. Were the predictor variables and the outcome evaluated in the whole sample selected initially?	No	Yes	Yes	No
A	6. Are the statistical methods used to construct and validate the rule clearly described?	Yes	Yes	Yes	Yes
B	7. Can the performance of the rule be calculated?	No	No	No	Yes
B	8. How precise was the estimate of the treatment effect?	No	Yes	No	Yes
C	9. Would the prediction rule be reliable and the results interpretable if used for your patient?	No	Yes	Yes	Yes
C	10. Is the rule acceptable in your case?	No	Yes	Yes	Can’t tell
C	11. Would the results of the rule modify your decision about the management of the patient or the information you can give to him/her?	No	Yes	Yes	Can’t tell
Overall Score	Percentage of ‘yes’ responses	27.3%	72.7%	72.7%	54.5%

^a ^Section A focuses on validity of study results and whether it is worth continuing; Section B focuses on the study results; Section C identifies the applicability of the results and findings

### Model development and presentation

The four studies used different modelling techniques to develop the predictive model including: likelihood basis pursuit [31]; repeated events survival model [30]; machine learning approaches using decision tree [29]; and random forest [32]. The model outcome measures in all studies were defined as a binary outcome (i.e., whether the client/resident experienced at least one fall), with two studies [29, 31] limiting the outcome assessment to the first three months of the study period. The number of predictors used in model development ranged from six [31] to over 130 [32]. The presentation of final models varied across studies. Two studies reported model output as probabilities based on the combination of variables in the model [31, 32]. Two studies developed risk categories; one was based on a decision tree [29] and the other based on a score which was then converted to a risk decile with the highest decile indicating the highest risk of a fall [30]. With the exception of Volrathongchai, Brennan [31] the other authors reported the rate of falls in their studies. Lo, Lynch [32] reported a 5.14% fall incidence rate. Two studies reported fall rates across their risk categories, ranging from 5.0–35.0% and 2.3–32.3% [29, 30].

### Model performance and evaluation

All studies except one [31] reported model performance in the derivation (training) [30, 32] and or validation (testing) sample [29, 30]. One study reported the Akaike Information Criterion (AIC) to compare the performance of four models in both derivation and validation cohorts [30]. Another used balanced accuracy to assess the accuracy of three models against a baseline model and found an accuracy of 0.51–0.62 depending on the model [32]. This study also utilised the area under the receiver operating characteristic curves (AUC), reporting values ranging from 0.60–0.67 [32]. One study conducted an external validation using samples from four different regions in Canada (Ontario, Manitoba, Alberta and British Colombia) and reported C-statistics (AUC) ranging from 0.55–0.60 [29] None of the studies reported the sensitivity and specificity of their predictive models.

### Synthesis: risk factor predictors

Many person-level predictors were used in the models (Table 3). We identified seven categories: (1) Demographics, (2) Assessments conducted with the client or resident, for example, cognitive performance scale, (3) Fall history, (4) Medication, (5) Health conditions, (6) Physical abilities and (7) Environmental factors.

**Table 3:** Characteristics of predictors used in the final models of included studies

**Predictors**	Volrathongchai et al. (2005) [31]	Marier et al. (2016) [30]				Kuspinar et al. (2019) [29]	Lo et al. (2019) [32]		
		1: MDS Assessments^A^	2: MDS Assessments & EMR only^A^	3: MDS assessments & EMR duplicates^A^	4: MDS assessments & EMR Only & EMR Duplicates^A^		1: Combined (OASIS & MACH)	2: Oasis Model	3: MACH Model
**Demographics**									
Age						**X**			
Sex						**X**			
**Assessments**									
Cognitive Performance Scale						**X**			
Activities of Daily Living Hierarchy						**X**			
Worsening of Activities of Daily Living Status						**X**			
Pain Scale						**X**			
Managing Medication						**X**			
Missouri Alliance for Home Care Fall Risk Assessment (MACH)							**X** ^b^		**X** ^b^
Outcome and Assessment Information Set (OASIS-C) – 46 Items^c^							**X**^c^	**X**^c^	
Unstable Health Patterns						**X**			
**Fall History**									
Fall in Last 30 Days	**X**	**X**	**X**	**X**	**X**				
Fall in 31–180 Days	**X**	**X**	**X**	**X**	**X**				
**Medication**									
Anticoagulant		**X**	**X**	**X**	**X**				
Anticonvulsant			**X**		**X**				
Antihypertensive (Alpha II Agonist)			**X**		**X**				
Antihypertensive (Alpha-Adregen Blocker)			**X**		**X**				
Antipsychotic (last 7 days)	**X**								
Antipsychotic		**X**	**X**	**X**	**X**				
Anxiolytic				**X**	**X**				
Antidepressant				**X**	**X**				
Diuretic		**X**	**X**	**X**	**X**				
Hypnotic		**X**	**X**	**X**	**X**				
Opioid Analgesic			**X**		**X**				
Psychotropic			**X**		**X**				
**Health Conditions**									
Anaemia		**X**	**X**	**X**	**X**				
Alzheimer's Disease		**X**	**X**	**X**	**X**				
Atrial Fibrillation		**X**	**X**	**X**	**X**				
Behavioural Problems				**X**	**X**				
Cognitive Impairment		**X**	**X**	**X**	**X**				
Depression		**X**	**X**	**X**	**X**				
Diagnosis Causing Imbalance		**X**	**X**	**X**	**X**				
Hearing loss	**X**								
Hemiplegia or Hemiparesis	**X**								
Incontinence				**X**	**X**	**X**			
Mental Instability		**X**	**X**	**X**	**X**				
Malnutrition		**X**	**X**	**X**	**X**				
Osteoporosis		**X**	**X**	**X**	**X**				
Pain		**X**	**X**	**X**	**X**				
Parkinson’s Disease						**X**			
Vision poor		**X**	**X**	**X**	**X**				
Urinary Tract Infection				**X**	**X**				
**Physical abilities**									
Ambulation				**X**	**X**				
Imbalance		**X**	**X**	**X**	**X**				
Mode of Expression: Writing	**X**								
Mobility in Bed						**X**			
Primary Mode of Locomotion						**X**			
Restricted Lower Range of Motion		**X**	**X**	**X**	**X**				
Use of Walking Aids		**X**	**X**	**X**	**X**				
Unsteady Gait						**X**			
Wandering				**X**	**X**				
Wheelchair Use									
**Environmental factors**									
Admission from Transfer		**X**	**X**	**X**	**X**				
Week after Admission				**X**	**X**				
Week after Room Change			**X**		**X**				
Total Number of Variables	**6**	**21**	**27**	**29**	**35**	**13**	**300 + 10**^**bc**^	**300**^**c**^	**10**^**b**^

*MDS* Minimum Data Set, *EMR* Electronic Medical record, *OASIS* Outcome and Assessment information Set, *MACH-10* Missouri Alliance for Home Care fall risk assessment

^a^ Models also contained the covariates: days since admission, days since admission squared, interactions between each risk factor and days since admission and duration of time that each resident exhibits a particular risk profile

^b^ MACH scale includes the following ten binary variables: Age 65 + , Diagnosis (three or more co-existing), Prior history of falls within 3 months, Incontinence, Visual impairment, Impaired functional mobility, Environmental hazards, Polypharmacy (four or more prescriptions – any type), Pain affecting level of function, and Cognitive impairment

^c^ OASIS contained 46 items of the 115, chosen based on literature and association with falls. These were used this to create 300 estimates. Example items included 2 + hospitalisations in the past year, shortness of breath, ability to hear and 2 + falls with an injury in the past year

## Discussion

Our review identified four studies reporting the development of nine predictive models using electronic health records in residential and home-based aged care services. These models were used to identify individuals receiving aged care services most likely to experience a fall in the near future based on factors identified through routinely collected data. Only one study conducted an external validation [29]. However, the limited information presented about the predictive performance of the identified models in this review means that they have limited utility for other organisations considering applying these models for people in their care.

### Using electronic health record data

Many risk factors for falls have been identified in the literature, with a strong predictor of a fall being a previous fall [10]. Whilst the use of electronic health data can provide access to an extensive set of variables and thus add to the accuracy of predictive models, only one study explored a model to predict a fall for first time fallers [29]. The predictors included in the models identified in this review varied substantially based on the modelling technique used, demonstrating the multifactorial nature of risk factors associated with a fall, however, none of the models were good at predicting a fall as indicated in the statistical model performance tests. Additionally, electronic health record data has limitations regarding the recording of falls, including being miscoded, the potential for missing data due to incidents of falls not being recorded (particularly for those that live at home receiving aged care services), and the scarce sharing of falls information data between health systems (such as hospitals and residential aged care).

### Predicting fall severity

To advance the field of real-time predictive fall risk modelling, it would be useful to not just predict a fall but also the potential outcome of that fall. For example, falls are often categorised as an injurious fall, a fall resulting in hospitalisation, or a non-injurious fall [33]. None of the studies included in the review explored the concept of injury impact or severity. By using these categories, providers would be able to highlight individuals at increased risk of a fall resulting in hospitalisation compared to individuals with a non-injurious fall and help tailor the appropriate interventions and resources required [9].

### Predictive model methods

One of the main methodological limitations of the studies included in this review was the use of sub-optimal statistical methods to develop the prediction models. It is important to consider falls as recurrent events as they can occur multiple times. Any statistical or machine learning methods used to predict falls should therefore account for the potential recurrence and correlation of the outcome data. Of the four studies in this review, only one study [30] utilised a method that is appropriate for modelling recurrent events (i.e., repeated events survival model). Although several suitable methods are currently available [34, 35], most fall-related studies utilise inappropriate statistical methods. In a systematic review by Donaldson et al. that included 83 fall prevention randomised controlled trials, only one-third of the trials utilised suitable statistical methods [35].

The choice of statistical methods to model recurrent events is dependent on the research question and the nature of the available data. If data on the time of the event are not of interest or not measured, Poisson or negative binomial models can be used [34]. On the other hand, if data on the time of the event are relevant, survival analysis-based approaches can be used. Most commonly used survival analysis-based approaches for recurrent events include the *Andersen-Gill*, *Wei, Lin and Weissfeld*, *Prentice-Williams-Peterson*, and *Frailty models*; all of which are extensions of the Cox proportional hazard model and implemented in standard statistical software packages including R, SAS and Stata [34, 36, 37]. Frailty models have the added advantage of incorporating random effects to account for certain unmeasured or unknown factors. If survival time is measured in discrete values (e.g., weeks to fall occurrence), *discrete time survival models* can be utilised [38].

Advanced methods such as *joint models* (techniques that allow simultaneous modelling of longitudinal and survival data) [39], *landmark models *[40], and machine learning based on deep learning approaches [41] have been utilised for dynamic prediction of recurrent events. If data are characterised by a multilevel (hierarchical) structure, statistical methods that account for both the potential correlation of recurrent outcomes and the clustering effect (that is, the potential correlation between outcomes of patients in the same facility) should be used. Examples of this may include using discrete time survival, frailty, joint or landmark models in a multilevel framework [39, 40, 42, 43].

### Implementing predictive models

Other sectors have demonstrated significant benefits from implementing predictive models for a wide range of conditions, including cerebrovascular and hypertensive diseases [44], diabetes [45], and nursing outcomes more generally [46]. Dashboards are one method that could be used for implementing risk models in aged care—dashboards have been used successfully in primary care settings to integrate information across data sources and present these data together to improve patient care [47]. Additionally, predictive models used in tandem with clinical decision support have been shown to improve patient outcomes [47] and should be considered when deploying risk models in aged care settings.

The combination of accurate and dynamic predictive models coupled with clinical decision support software and implementation of evidence-based strategies to prevent falls has the potential to substantially reduce the rate of falls and fall-related injury in some of the most vulnerable members of our society. Combining accurate predictive models with implementation of evidence-based strategies to reduce falls [9] would equip aged care staff with adequate information and resources to reduce falls.

## Implications of findings

Future research should focus on using optimal statistical techniques when developing predictive models in RAC by considering a fall as a recurrent event and accounting for potential reoccurrence and correlation of the outcome data. End-user engagement during development phases of these predictive models would ensure that resulting models are relevant and usable by those monitoring and treating falls in older people. Whilst predictive models hold great potential for identifying risk in real-time, the implementation and evaluation of these models within aged care services is critical to determine their true effectiveness and cost-effectiveness for health and wellbeing outcomes. This would provide pivotal evidence for policy makers to make decisions around the need for future predictive models in RAC, exploring other adverse outcomes as well.

### Limitations and strengths

This systematic review has several limitations. Firstly, the review was limited to studies published in English, and therefore, we may have missed some predictive models for falls published in other languages. Secondly, there may be predictive models for falls in those receiving aged care services that have been published in the grey literature, which would have been missed by our search. Thirdly, the limited availability of research on this topic resulted in an inability to pool results. Lastly, we found inconsistent terminology was used to describe fall risk models in the literature, meaning we might have missed articles using different terminology, though we had clinical expertise within our authorship team and we consulted a clinical librarian regarding our search strategy to minimise this. The strengths of the systematic review included adhering to the PRISMA guidelines, using a broad search strategy to ensure all articles were captured, and a rigorous screening process.

## Conclusions

Large amounts of data are collected and stored electronically during day-to-day routine practice by aged care services. These data could be used to predict individuals at risk of falls and help guide interventions to lessen fall risk. We systematically reviewed the literature on predictive models for falls using electronic health records of individuals receiving residential, home or community aged care services. Our systematic review represents the limited contemporary evidence on predictive models for falls risk in aged care services, highlighting the need for more research and robust statistical methods applied to falls predictive models.

## Supplementary Information

**Additional file 1.** 

**Additional file 2.** 

**Additional file 3.** 

## Data Availability

Not applicable.

## Abbreviations

AIC: Akaike Information Criteria
AUC: Area under the curve
CASP: Critical Appraisal Skills Program
EHR: Electronic health record
EMR: Electronic Medical record
LBP: Likelihood Basis Pursuit
MDS: Minimum Data Set
MACH-10: Missouri Alliance for Home Care fall risk assessment
NR: Not reported
OASIS: Outcome and Assessment information Set
RAI-HC: Resident Assessment Instrument-Home Care
