# Supplementary material for: The use of predictive fall models for older adults receiving aged care, using routinely collected electronic health record data: a systematic review
Source: BMC Geriatr. 2022 Mar 16;22:210. doi: 10.1186/s12877-022-02901-2 (PMC8923829; doi:10.1186/s12877-022-02901-2)
Supplement: Supplementary file 2 — Additional file 2. [file 12877_2022_2901_MOESM2_ESM.docx]

**SYSTEMATIC REVIEW PROTOCOL**

**Title: The use of predictive fall models for older adults receiving aged care, using routinely collected electronic health record data: A systematic review**

**Review team:**

MQ team: Karla Seaman, Kristiana Ludlow, Laura Dodds, Mikaela Jorgensen, Joyce Siette, Johanna Westbrook (Student placements: Annaliese Eymael, Caroline Lin), Nasir Wabe, Amy Nguyen

Working Group: Stephen Lord, Jacqueline Close, Libby O'Toole,

**Research questions:**

*What fall models have been developed for use in home-based, community and residential aged care?*

**PICOS (Population, Intervention, Comparison, Intervention, Study Design) statement**

*Population:* Older adults in residential aged care, the community, or receiving formal care at home (>65 years old)

*Intervention:* Fall model

*Comparator:* No fall model

*Outcome:* Falls

*Study design:* No limits on study design. Could include models that have been developed or models that have been used in practice.

**Databases:**

Databases to be included in your search, i.e. Embase, Medline, etc. Databases to be searched include Cochrane Library, CINAHL Plus with Full Text, MEDLINE, Scopus, Web of Science with article inclusion from 2000 to the present.

For each of your PICO, what search terms will you use.

| **Population**: | **Intervention**: | **Comparator**: | **Outcome**: |
| --- | --- | --- | --- |
| Aged care | Model* |  | Fall* |
| Assisted living facilit* | Algorithm |  |  |
| Community based aged care | Screen* |  |  |
| Community based care | Predict* |  |  |
| Community care |  |  |  |
| Community support |  |  |  |
| Elder care |  |  |  |
| Home care |  |  |  |
| Home-based care |  |  |  |
| Home health care |  |  |  |
| Home support |  |  |  |
| Housing for the elderly |  |  |  |
| Long term care |  |  |  |
| Nursing care facilit* |  |  |  |
| Nursing home* |  |  |  |
| Old age home |  |  |  |
| Older adult* |  |  |  |
| Residential care |  |  |  |
| Residential facilit* |  |  |  |
| Skilled nursing facilit* |  |  |  |
| Social care |  |  |  |

| **Keywords** | | | |
| --- | --- | --- | --- |
| *MEDLINE* | Residential facilities/ assisted living facilities/ home for the aged/ nursing homes OR  Home care services/ home health nursing/ senior centres OR Health services for the aged/ OR  Adult day care centres/ OR | Models, statistical  OR  Algorithms/ or artificial intelligence/ or machine learning/ | Accidental falls |
| *CINAHL* | Nursing home OR  Nursing home patients OR  Housing for the elderly OR  Home health care OR  Skilled nursing facility OR  Residential care OR  Long term care OR  Older adult care OR  Home nursing, professional | Models, statistical OR  Algorithms OR  Artificial intelligence OR  Machine learning OR  Deep learning OR Prediction models | Accidental falls OR  Fall risk |
| *Cochrane* | Home care services OR Residential facilities OR  Long term care OR | Models, statistical OR  Algorithms OR  Machine learning | Accidental falls |
| *Scopus:* N/A | N/A | | |
| *Web of Science:* | N/A | | |

* Indicates truncation, e.g., “predict*” will capture “predict”, “predictive”, “predicted” etc.

**Definitions**

- *Older adult:* Person aged >65 years.
- *Residential aged care:* Long-term institutionalised care for older adults proving nursing care, assistance with daily living activities and psychosocial care, also referred to as nursing homes, skilled nursing facilities or assisted living facilities depending on country and context.
- *Home-based aged care:* Formal aged care services provided in a client’s own home.
- *Community-based aged care:* Care provided in community settings, e.g., day centres. Community-based and home-based aged care are sometimes used interchangeable in the literature.
- *Fall:* An event that results in a person coming to rest inadvertently on the ground or floor or other lower level.
- *Risk model:* A risk model is a statistical procedure for assigning an individual a probability of developing a future adverse outcome in a given time period. Our review specifically looks at the uses of real-time routinely collected data to predict falls risk. Tools administered to consumers to measure a static risk of fall, or interventions to prevent falls, are not considered risk models.

**Search strategy**

“Assisted living facilit*” OR “Community care” OR “Elder care” OR “Home care” OR “Housing for the elderly” OR “Long-term care” OR “Nursing care facilit*” OR “Nursing home*” OR “Old age home” OR “Older adult*” OR “Residential care” OR “Residential facilit*” OR “Skilled nursing facilit*”

AND

Fall*

AND

“model*” OR “predict*” OR “algorithm” OR “screen*”

***Limits***

Date: 2000-present

Language: English

**Date of search:**

**Final search: 24/07/20 – Took out the MesH term “aged” and the terms “screen” and “social care”**

| **Database** | **Results** |
| --- | --- |
| **MEDLINE (Ovid)** | **2,482** |
| **CINAHL** | **1,815** |
| **Scopus** | **3,721** |
| **Web of Science Core Collection** | **4,218** |
| **Cochrane Library** | **194** |
| **Total before duplicates removed** | **12,430** |

**Additional search: 24/07/20 – Added “social care”, “older people” and “care home” to search terms (numbers exclude all other residential aged care terms as this search adds to the previous one (third search) that included these terms**

| **Database** | **Results** |
| --- | --- |
| **MEDLINE (Ovid)** | **800** |
| **CINAHL** | **519** |
| **Scopus** | **1,211** |
| **Web of Science Core Collection** | **1,612** |
| **Cochrane Library** | **145** |
| **Total before duplicates removed** | **4,287** |

| **Database** | **Search field** | **Keywords/MeSH terms** | **Limits** | **Results: both settings** | |  |
| --- | --- | --- | --- | --- | --- | --- |
| CINAHL Plus with Full Text | N/A | Nursing home  Nursing home patients  Housing for the elderly  Home health care  Skilled nursing facility  Residential care  Long term care  Older adult care  Home nursing, professional  Accidental falls  Fall risk  Models, statistical | Language: English  Date: 2000-2020 | 2,334 | |  |
| MEDLINE complete | Abstract | *MH Exact subject heading:*  Nursing homes  Assisted living facilities  Home care services  Home care  Accidental falls  Models, Statistical | Language: English  Date: 2000-2020 | 3282 | |  |
| Scopus | Title, abstract, keywords | N/A – included in search field | Language: English  Date: 2000-present | 4,932 | |  |
| Web of Science Core Collection | Topic | N/A – included in search field | Language: English  Date: 2000-2020 | 5,830 | |  |
| Cochrane Library | Title, abstract, keywords | N/A – included in search field | Date: 2000-2020  (Language not applicable) | 339 | |  |
| **Total before duplicates removed** | | | | | 16,717 | |

**Inclusion criteria**

English language

Empirical studies published in peer-reviewed journals

Published 2000 onward

Involves older adults in residential, community, or home-based care settings

Includes fall models

Full-text available

**Exclusion criteria**

Language other than English

Grey literature, opinion/think pieces, studies published in non-peer-reviewed journals

Published prior to 2000

Older adults in acute or rehabilitation setting

Does not include fall models

Full-text not available

**Checking other Similar Systematic review registered and or published**

Checked Prospero on 1^st^ June 2020, Cochrane 1^st^ June, checked pubmed 1^st^ June, Checked JBI 28^th^ May 2020,
