# Supplementary material for: The use of predictive fall models for older adults receiving aged care, using routinely collected electronic health record data: a systematic review
Source: BMC Geriatr. 2022 Mar 16;22:210. doi: 10.1186/s12877-022-02901-2 (PMC8923829; doi:10.1186/s12877-022-02901-2)

| MEDLINE (Ovid) | | | | | |
| --- | --- | --- | --- | --- | --- |
| **Population** | | **AND Outcome** | | **AND Intervention** | |
| **Title** | "Aged care" OR "Assisted living facilit*" OR "Community based aged care" OR "Community based care" OR "Community care" OR "Community support" OR "Elder care" OR "Home care" OR "Home based care" OR "Home health care" OR "home healthcare" OR "Home support" OR "Housing for the elderly" OR "In-home care" OR "Long term care" OR "Nursing care facilit*" OR "Nursing home*" OR "Old age home" OR "Older adult*" OR "Residential care" OR "Residential facilit*" OR "Skilled nursing facilit*" OR "Social care" | **Title** | Fall* | **Title** | "Algorithm*" OR "Artificial intelligence" OR "Deep learning" OR "Machine learning" OR "Model*" OR "Predict*" OR "Screen*" |
| **OR Abstract** | "Aged care" OR "Assisted living facilit*" OR "Community based aged care" OR "Community based care" OR "Community care" OR "Community support" OR "Elder care" OR "Home care" OR "Home based care" OR "Home health care" OR "home healthcare" OR "Home support" OR "Housing for the elderly" OR "In-home care" OR "Long term care" OR "Nursing care facilit*" OR "Nursing home*" OR "Old age home" OR "Older adult*" OR "Residential care" OR "Residential facilit*" OR "Skilled nursing facilit*" OR "Social care" | **OR Abstract** | Fall* | **OR Abstract** | "Algorithm*" OR "Artificial intelligence" OR "Deep learning" OR "Machine learning" OR "Model*" OR "Predict*" OR "Screen*" |
| **OR MeSH terms** | Residential facilities/ or assisted living facilities/ or home for the aged/ or nursing homes OR  Home care services/ or home health nursing/ or senior centres OR  Health services for the aged/ OR  Adult day care centres/ | **OR MeSH terms** | Accidental falls | **OR MeSH terms** | Models, statistical  OR  Algorithms/ or artificial intelligence/ or machine learning/ |
| **Limits** | English language  2000-current | **Limits** | English language  2000-current | **Limits** | English language  2000-current |


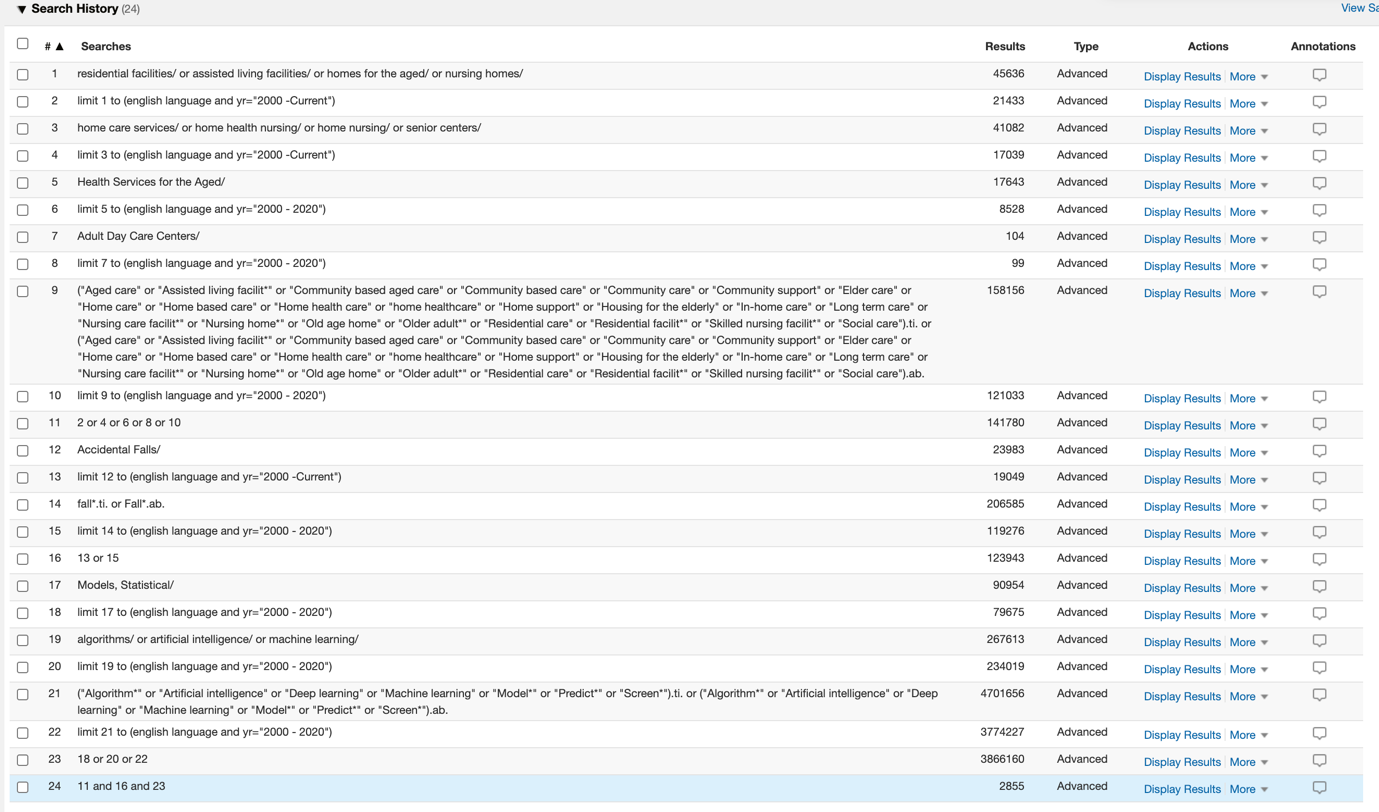


| Web of Science Core Collection | | | | | |
| --- | --- | --- | --- | --- | --- |
| **Population** | | **AND Outcome** | | **AND Intervention** | |
| **Topic** | "Aged care" OR "Assisted living facilit*" OR "Community based aged care" OR "Community based care" OR "Community care" OR "Community support" OR "Elder care" OR "Home care" OR "Home based care" OR "Home health care" OR "home healthcare" OR "Home support" OR "Housing for the elderly" OR "In-home care" OR "Long term care" OR "Nursing care facilit*" OR "Nursing home*" OR "Old age home" OR "Older adult*" OR "Residential care" OR "Residential facilit*" OR "Skilled nursing facilit*" OR "Social care" | **Topic** | Fall* | **Topic** | "Algorithm*" OR "Artificial intelligence" OR "Deep learning" OR "Machine learning" OR "Model*" OR "Predict*" OR "Screen*" |
| **Limits** | English language  2000-2020 | **Limits** | English language  2000-2020 | **Limits** | English language  2000-2020 |


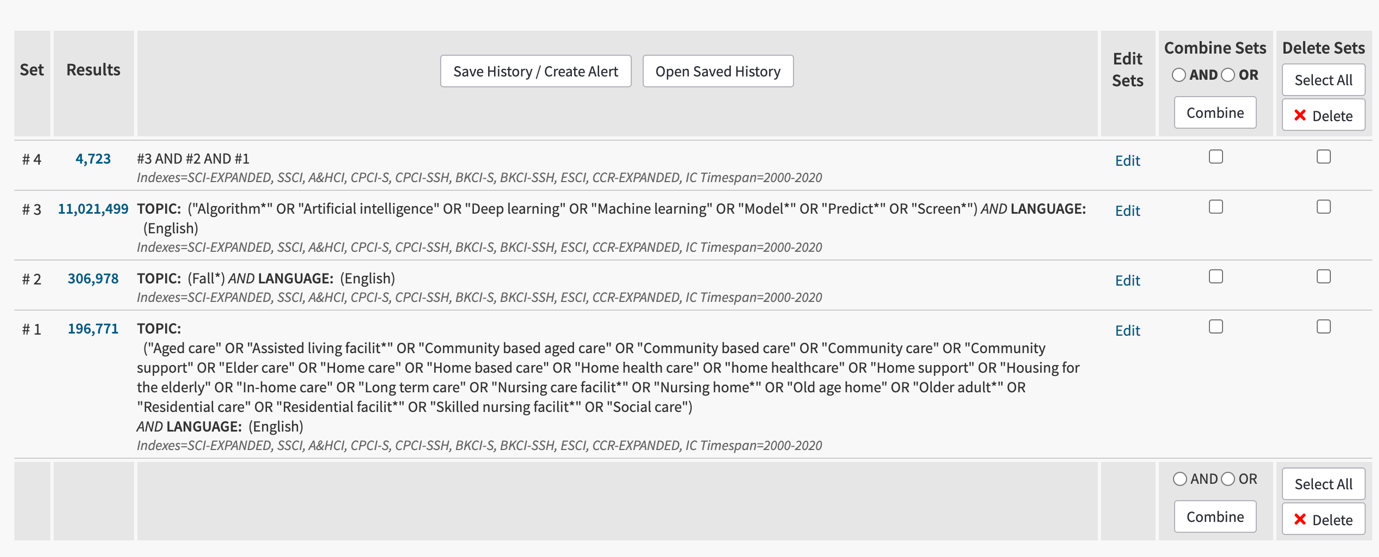


| Scopus | | | | | |
| --- | --- | --- | --- | --- | --- |
| **Population** | | **AND Outcome** | | **AND Intervention** | |
| **Topic** | "Aged care" OR "Assisted living facilit*" OR "Community based aged care" OR "Community based care" OR "Community care" OR "Community support" OR "Elder care" OR "Home care" OR "Home based care" OR "Home health care" OR "home healthcare" OR "Home support" OR "Housing for the elderly" OR "In-home care" OR "Long term care" OR "Nursing care facilit*" OR "Nursing home*" OR "Old age home" OR "Older adult*" OR "Residential care" OR "Residential facilit*" OR "Skilled nursing facilit*" OR "Social care"  (Note: terms had to be split over two searches due to limit on number of search terms). | **Topic** | Fall* | **Topic** | "Algorithm*" OR "Artificial intelligence" OR "Deep learning" OR "Machine learning" OR "Model*" OR "Predict*" OR "Screen*" |
| **Limits** | English language  2000-present | **Limits** | English language  2000-present | **Limits** | English language  2000-present |

( ( TITLE-ABS-KEY ( "Aged care"  OR  "Assisted living facilit*"  OR  "Community based aged care"  OR  "Community based care"  OR  "Community care"  OR  "Community support"  OR  "Elder care"  OR  "Home care"  OR  "Home based care"  OR  "Home health care"  OR  "home healthcare"  OR  "Home support" )  OR  TITLE-ABS-KEY ( "Housing for the elderly"  OR  "In-home care"  OR  "Long term care"  OR  "Nursing care facilit*"  OR  "Nursing home*"  OR  "Old age home"  OR  "Older adult*"  OR  "Residential care"  OR  "Residential facilit*"  OR  "Skilled nursing facilit*"  OR  "Social care" ) )  AND  PUBYEAR  >  1999 )  AND  ( TITLE-ABS-KEY ( fall* )  AND  PUBYEAR  >  1999 )  AND  ( TITLE-ABS-KEY ( "Algorithm*"  OR  "Artificial intelligence"  OR  "Deep learning"  OR  "Machine learning"  OR  "Model*"  OR  "Predict*"  OR  "Screen*" )  AND  PUBYEAR  >  1999 )  AND  ( LIMIT-TO ( LANGUAGE ,  "English" ) )


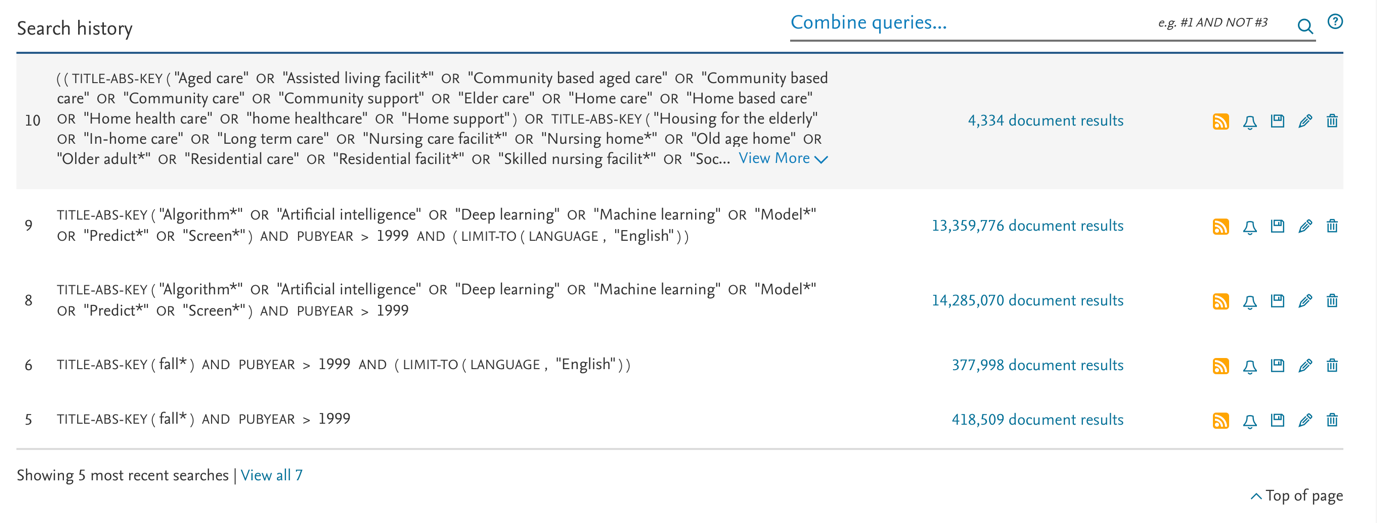


| CINAHL | | | | | |
| --- | --- | --- | --- | --- | --- |
| **Population** | | **AND Outcome** | | **AND Intervention** | |
| **Title** | "Aged care" OR "Assisted living facilit*" OR "Community based aged care" OR "Community based care" OR "Community care" OR "Community support" OR "Elder care" OR "Home care" OR "Home based care" OR "Home health care" OR "home healthcare" OR "Home support" OR "Housing for the elderly" OR "In-home care" OR "Long term care" OR "Nursing care facilit*" OR "Nursing home*" OR "Old age home" OR "Older adult*" OR "Residential care" OR "Residential facilit*" OR "Skilled nursing facilit*" OR "Social care" | **Title** | Fall* | **Title** | "Algorithm*" OR "Artificial intelligence" OR "Deep learning" OR "Machine learning" OR "Model*" OR "Predict*" OR "Screen*" |
| **OR Abstract** | "Aged care" OR "Assisted living facilit*" OR "Community based aged care" OR "Community based care" OR "Community care" OR "Community support" OR "Elder care" OR "Home care" OR "Home based care" OR "Home health care" OR "home healthcare" OR "Home support" OR "Housing for the elderly" OR "In-home care" OR "Long term care" OR "Nursing care facilit*" OR "Nursing home*" OR "Old age home" OR "Older adult*" OR "Residential care" OR "Residential facilit*" OR "Skilled nursing facilit*” OR “social care” | **OR Abstract** | Fall* | **OR Abstract** | "Algorithm*" OR "Artificial intelligence" OR "Deep learning" OR "Machine learning" OR "Model*" OR "Predict*" OR "Screen*" |
| **OR Subject terms** | Nursing home OR  Nursing home patients OR  Housing for the elderly OR  Home health care OR  Skilled nursing facility OR  Residential care OR  Long term care OR  Older adult care OR  Home nursing, professional OR | **OR Subject terms** | Accidental falls OR  Falls risk | **OR Subject terms** | Models, statistical OR  Algorithms OR  Artificial intelligence OR  Machine learning OR  Deep learning |
| **Limits** | English language  2000-2020 | **Limits** | English language  2000-2020 | **Limits** | English language  2000-2020 |


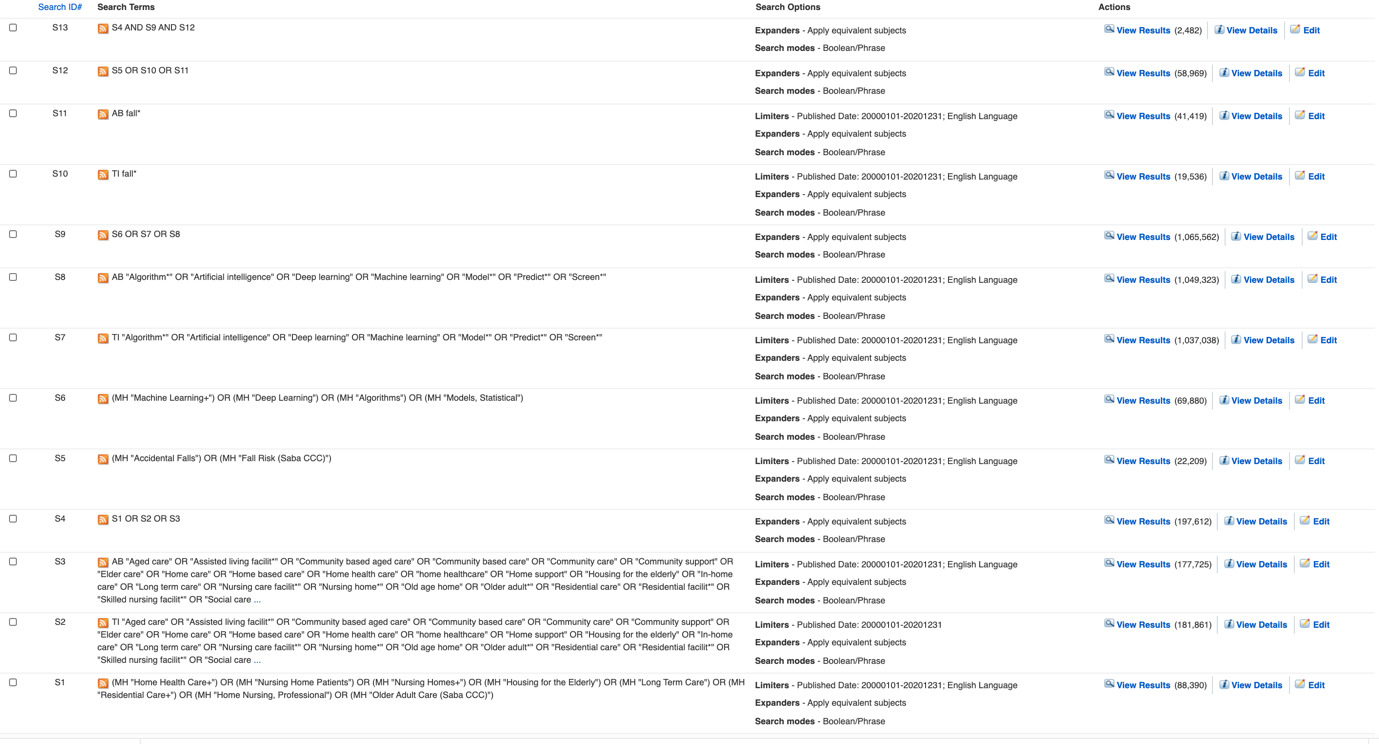


| Cochrane | | | | | |
| --- | --- | --- | --- | --- | --- |
| **Population** | | **AND Outcome** | | **AND Intervention** | |
| **Title** | "Aged care" OR "Assisted living facilit*" OR "Community based aged care" OR "Community based care" OR "Community care" OR "Community support" OR "Elder care" OR "Home care" OR "Home based care" OR "Home health care" OR "home healthcare" OR "Home support" OR "Housing for the elderly" OR "In-home care" OR "Long term care" OR "Nursing care facilit*" OR "Nursing home*" OR "Old age home" OR "Older adult*" OR "Residential care" OR "Residential facilit*" OR "Skilled nursing facilit*" OR "Social care" | **Title** | Fall* | **Title** | "Algorithm*" OR "Artificial intelligence" OR "Deep learning" OR "Machine learning" OR "Model*" OR "Predict*" OR "Screen*" |
| **OR Abstract** | "Aged care" OR "Assisted living facilit*" OR "Community based aged care" OR "Community based care" OR "Community care" OR "Community support" OR "Elder care" OR "Home care" OR "Home based care" OR "Home health care" OR "home healthcare" OR "Home support" OR "Housing for the elderly" OR "In-home care" OR "Long term care" OR "Nursing care facilit*" OR "Nursing home*" OR "Old age home" OR "Older adult*" OR "Residential care" OR "Residential facilit*" OR "Skilled nursing facilit*" OR "Social care" | **OR Abstract** | Fall* | **OR Abstract** | "Algorithm*" OR "Artificial intelligence" OR "Deep learning" OR "Machine learning" OR "Model*" OR "Predict*" OR "Screen*" |
| **OR Subject terms** | Home care services  Residential facilities OR  Long term care OR | **OR Subject terms** | Accidental falls | **OR Subject terms** | Models, statistical OR  Algorithms OR  Machine learning OR |
| **Limits** | English language  2000-2020 | **Limits** | English language  2000-2020 | **Limits** | English language  2000-2020 |


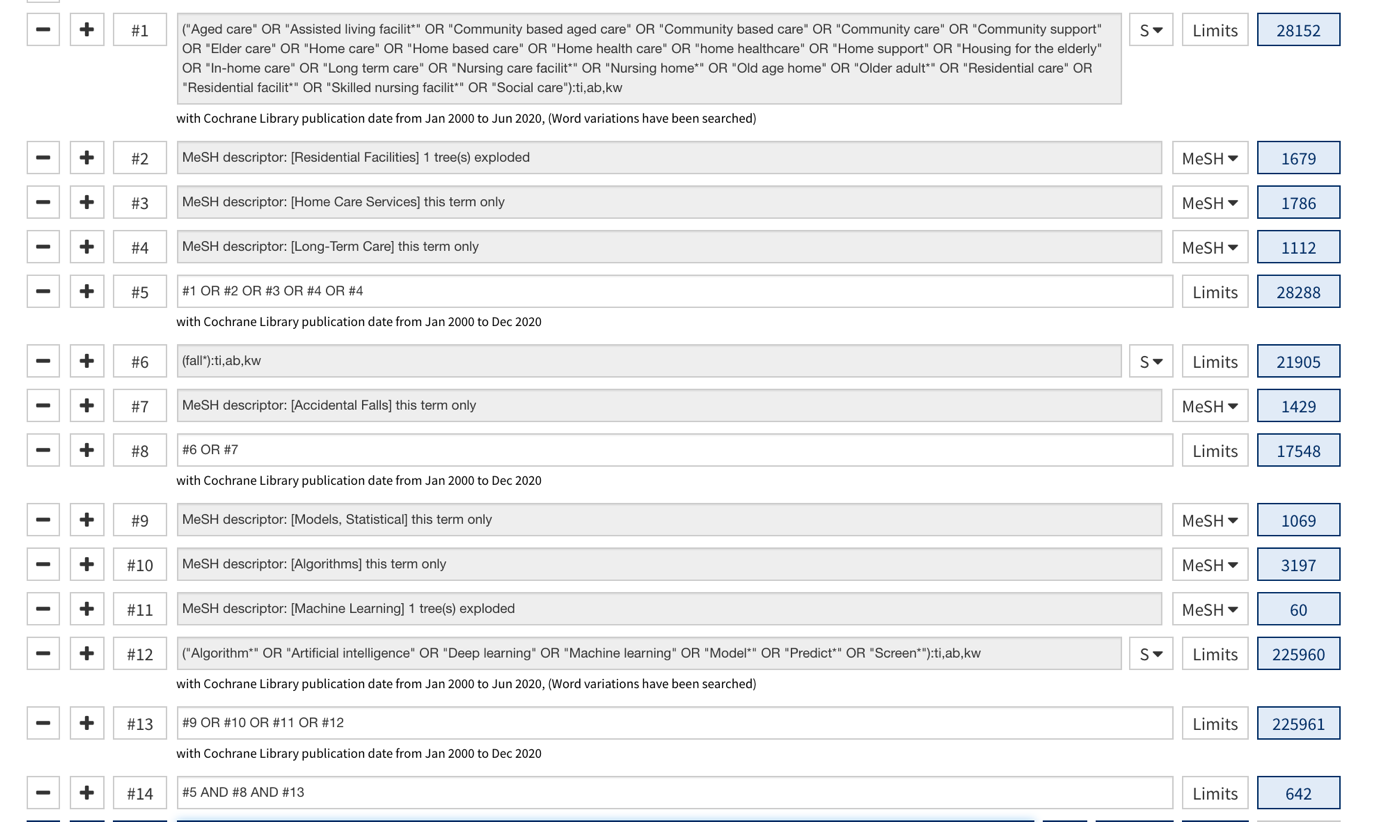

Supplement: Supplementary file 3 — Additional file 3. [file 12877_2022_2901_MOESM3_ESM.docx]
